# Contrasting Screen-Time and Green-Time: A Case for Using Smart Technology and Nature to Optimize Learning Processes

**DOI:** 10.3389/fpsyg.2018.00773

**Published:** 2018-06-01

**Authors:** Theresa S. S. Schilhab, Matt P. Stevenson, Peter Bentsen

**Affiliations:** ^1^Future Technology, Culture and Learning, Danish School of Education, University of Aarhus, Copenhagen, Denmark; ^2^Center for Outdoor Recreation and Education, The Forest and Landscape College, University of Copenhagen, Fredensborg, Denmark; ^3^Steno Health Promotion Research, Steno Diabetes Center Copenhagen, Capital Region, Gentofte, Denmark

**Keywords:** attention, attention restoration theory, closed signification, creative thinking, divergent thinking, learning, mind wandering, mobile technology

## Introduction

Tablets and smartphones (i.e., mobile technology) as learning tools for school use is on the rise worldwide (Norris and Soloway, 2015). The technology is reported to impact positively on learning outcomes (Major et al., 2017), by facilitating contextual and situated learning (Brown and Mbati, 2015). For instance, mobile devices are thought to stimulate personalized and informal learning by corroborating and adapting to the interests, preferences, and competencies of learners (Traxler and Wishart, 2011), while affording personal publishing and sharing (Mbati, 2017).

However, exposure to screens may also have more undesirable side-effects of concern to formal and informal learning. In so-called iPad schools where books are switched for iPads in class, play during break-time shifts from physical to more sedentary activities (Schilhab, 2017a). Crudely put, engagement with the external world of concrete phenomena and spontaneous events is switched for engagement with the mediated world of smart technology, where children watch and share YouTube videos, read Wikipedia, and are exposed to vast amounts of information from others (Holloway et al., 2013; Duarte Torres et al., 2014).

Hence, along with the increased use of mobile technology come attentional and cognitive shifts pertaining to the learning and development of the individual (e.g., Ward et al., 2017). Numerous studies have demonstrated that smart technology use influences attentional and cognitive processes in unexpected ways. For instance, it has been reported that devoting attention to mobile phones voluntarily or involuntarily changes the content and dynamics of conversations (Turkle, 2015), resulting in shallower content (Przybyliski and Weinstein, 2013) and lower levels of reported empathic concerns among interlocutors (Misra et al., 2016). It has also been argued that smart technology's capacity as information store has profound consequences on how we manipulate and memorize learned material (Sparrow et al., 2011; Barr et al., 2015; Dong and Potenza, 2017), although the actual effects on learning are also disputed (Aagaard, 2015; Heersmink, 2016). In meta-cognitive research, on-screen readers performed worse than print readers when tested in connection with self-regulated reading of expository texts (Ackerman and Goldsmith, 2011), suggesting that screen reading alters the recruitment of mental efforts (Lauterman and Ackerman, 2014).

In comparison, the natural world seems to engage attentional and cognitive processes differently. Following Attention Restoration Theory (ART, e.g., Kaplan, 1995) in opposition to screen watching (e.g., television), *unthreatening* greenish outdoor environments typically accessible to both urban and country dwellers stimulate by so-called soft fascination (Kaplan and Berman, 2010). Please note that *threatening* greenish outdoor environments may have more intrusive, yet desirable cognitive effects (e.g., Kahn et al., 2009). Accordingly, resting in green environments enhances so-called executive functioning (Bratman et al., 2012) in use when concentrating and thinking, and is therefore central for academic success (Diamond, 2013). Arguments for exposing students to nature are partly based on this effect (Matsuoka, 2010; Kuo et al., 2017). Although the restorative effect of soft nature on cognitive functioning, as proposed by ART, is persuasive with respect to promoting nature interventions in school, another much more profound effect of relevance to success in school and life not addressed by ART has gone largely unnoticed.

We advocate that the mental work occurring *during* restoration of executive functioning, so-called mind wandering, e.g., off-task thoughts that occur either with or without intention (Smallwood and Schooler, 2006), is crucially important in its own right. Given that screen watching and screen use is more likely to affect attentional and cognitive processes by hard fascination (Kaplan and Berman, 2010), to an extent that sometimes renders mobile technology use addictive (e.g. Rosen et al., 2013; Billieux et al., 2015), thus tapping into self-regulatory processes (Schilhab, 2017b), nature's facilitating effect on mind wandering becomes noteworthy.

In what follows, we (a) highlight how nature-induced soft fascination leaves room for spontaneous thoughts, which are under increased pressure from the mobile technology-induced hard fascination and more controlled thoughts and (b) emphasize the need for research relating green environments, open monitoring and divergent thinking.

## Attention

Forming part of executive functions (Engle, 2002; Posner et al., 2013), attentional control is closely related to success in school (Diamond, 2011). James (1892) famously distinguished between *involuntary* and *voluntary* attention, also known as stimulus-dependent and directed attention (e.g., Chun et al., 2011). The former refers to attention that requires no effort, such as when something dangerous, pleasurable or novel occurs (e.g., Sood and Jones, 2013) whereas the latter refers to the kind of attention employed when something is not particularly interesting and therefore requires a good deal of mental effort (Kaplan and Berman, 2010). Thus, stimulus-dependent attention often depends on external sense activity that drives learning automatically and *bottom-up*, whereas directed attention is independent of stimulus characteristics and works *top-down* (Wilson, 2002).

As noted by Kaplan and Berman (2010), James (1892, p. 88) pointed to “strange things, moving things, wild animals, bright things, pretty things, metallic things, words, blows, blood, etc. etc. etc.” as engaging stimulus-dependent attention. In this understanding, mobile technology seems entirely unmatched in its ability to “call” up the attention of its user. Mobile technology affords immediate access to pleasure, and unexpected and novel stimuli and thus taps heavily into our attentional resources (Lee et al., 2014; Li et al., 2015) combatting e.g. social anxiety and boredom (Elhai et al., 2017) or feeding attentional impulsiveness (Roberts et al., 2015). Even long-term attentional effects, the so-called phantom vibration and phantom ringing hallucinations, seem to occupy the mind of heavy mobile technology users (Lee et al., 2014; Tanis et al., 2015).

## Nature-induced soft fascination

Conversely, natural stimuli seem to capture attentional processes in an opposing way, although it is worth noting that “untrammeled” and unmanaged wild nature is likely to have different attentional effects (Davis and Gatersleben, 2013). ART suggests that non-threatening natural environments are experienced with less cognitive effort, because they are “softly fascinating” with no elements that compete with each other for attentional focus (Kaplan, 1995). ART predicts that perceiving natural stimuli will allow finite cognitive capacities, such as focusing attention, to restore, alleviating the individual from cognitive fatigue that is experienced when these capacities are overused (Kaplan, 1995; Berman et al., 2008). Indeed, there is an existing research base that supports the notion that exposure to nature can be beneficial to cognitive processes (for review, see Ohly et al., 2016).

We suggest that non-threatening natural environments that softly fascinate have positive effects on cognition through the facilitation of spontaneous thought processes.

According to ART, nature-bound stimuli are less likely to signify a sense of immediate danger or otherwise pull attention along particular thought paths. Hence, engaging with nature-bound stimuli involves comparably fewer symbolic associations than engaging with smart technology. A pond full of carp signifies nothing or very little beyond itself. Carp swimming just “are”—the observation does not trigger a sense of danger, hard fascination difficult to disengage from or intentions to act, whereas a picture of carp as in advertisements normally signifies or stands for something different that instigates serial thought processes calling upon directed attention. It is likely that the “closed signification,” which is the fact that nature's stimuli point to themselves and not away from themselves to something beyond, provides nature with the strength to decelerate or even obliterate thought processes (Schilhab, 2017c).

In a study that illustrated how the brain processes natural and non-natural stimuli differently, Berto et al. (2008) used eye-tacking technology to investigate how participants viewed two types of scenes. They found that viewing natural scenes was associated with greater exploration and fewer fixations; however, when viewing urban scenes, participants were more likely to fixate on certain stimuli. Greater scene exploration suggests greater fascination that is not cognitively demanding, whereas frequent and longer fixation suggests that attention is more readily captured by these stimuli that they are more cognitively demanding to process (Berto et al., 2008).

Being in a safe natural environment, where the surrounding stimuli have no intrinsic threat, goal, or task associated with them, may benefit non-perceptual cognitive processes important for learning. An environment with no goal-directed or task-positive stimuli may also be associated with activation of task-unrelated neural networks, such as the default mode network (DMN) (Andrews-Hanna et al., 2014). The DMN is associated with autobiographical memory and mind-wandering and has been shown to be separately involved in the maintenance phase of working memory alongside task-positive networks (Piccoli et al., 2015). Maintaining and remembering newly acquired information is one of the most commonly demonstrated cognitive benefits of exposure to natural stimuli (Ohly et al., 2016). Moreover, the cognitive load in working memory tasks can be predictive of the impact of natural stimuli, where the harder the task conditions, the greater the cognitive restoration associated with exposure to nature (e.g., Dadvand et al., 2015). This suggests that the harder the brain is working to shield memorized information from external and internal distraction, the greater the impact a natural environment will have on restoration of this and associated abilities.

## Divergent thinking

The reduced pull on thought processes facilitates more self-generated thoughts where the mind “move[s] hither and thither without fixed course or certain aim” (Christoff et al., 2016, p. 719). Such episodes are considered adaptive since they allow individuals to, for instance, prepare for future events, to sustain a sense of self-identity and to re-interpret social encounters (Andrews-Hanna et al., 2014).

Spontaneous thought processes associated with the reduced external pull on thoughts also loosely resemble divergent thinking processes stimulating creative thinking and abilities to think “out of the box” (Colzato et al., 2012)^1^.

This might suggest that in contrast to smart technology use, nature-bound stimuli are more likely to endorse so-called open monitoring mind states prevalent in certain meditative traditions (Tang and Posner, 2009; Howell et al., 2011; Lebuda et al., 2016). Following Hommel and Colzato (2017), focused-attention meditation (FAM) differs from open-monitoring meditation (OMM) and have different and sometimes even opposite impacts on cognitive processes. Whereas FAM traditionally trains directed attention capacities by sustained attention on a specific object, OMM “sustains attentive monitoring of anything that might occur in experience without focusing on any explicit object” (ibid. p. 115; see also Lutz et al., 2008).

In the current context, we suggest that nature-bound stimuli are likely to induce open-monitoring mental states that typically promotes the divergent thinking style that allows many new ideas to be generated (Leong et al., 2014; Colzato and Hommel, 2017; Colzato et al., 2017). Studies examining the impact of acute moderate and intense physical exercise on convergent and divergent thinking in athletes and non-athletes (S Colzato et al., 2013) or the effect of walk (Keinänen, 2016; Zhou et al., 2017) could form the backdrop for a future research design to test the impact of nature-bound vs. mobile technology-bound stimulation (for the distinction between the effect of the outdoors and physical activity, see Oppezzo and Schwartz, 2014).

## Conclusions and suggestions for future research

Based on these ideas, we suggest that exposure to a natural environment or natural stimuli, may be seen as a useful and relevant intervention strategy to counteract the effect of exhausted cognitive capacities associated with overuse of smart technology. Coupling periods of smart technology use with periods of exposure to a natural environment may be optimal for learning.

Given the increasing use of mobile technology worldwide we also need to identify how technology can be used to encourage more children to get outside (Schilhab, 2018). The consequences of using smart technology within natural environments are not yet known although new research on the effect of Pokémon Go may provide some early indications (LeBlanc and Chaput, 2017; Ruiz-Ariza et al., 2018). Thus, future studies should investigate both how natural stimuli may counteract exhausted cognitive capacities and the effects of mixing nature-bound and technology-bound stimulations when mobile technologies are used in nature experiences.

## Author contributions

TS, MS, and PB: conceived of the study; TS and MS: contributed conception of the study; TS: wrote the first draft of the manuscript; MS: wrote sections of the manuscript. All authors contributed to manuscript revision, read and approved the submitted version.

### Conflict of interest statement

The authors declare that the research was conducted in the absence of any commercial or financial relationships that could be construed as a potential conflict of interest.

## Footnotes

^1^For a straightforward description of divergent and convergent thinking see Jones and Estees (2015, p. 474) who define divergent thinking tasks as generally focused on generating several possible “imaginative” answers, whereas convergent thinking tasks generally entail a narrowing of possible solutions to one optimal answer.

## References

[B1] Aagaard J. (2015). Media multitasking, attention, and distraction: a critical discussion. Phenomenol. Cogn. Sci. 14, 885–896. 10.1007/s11097-014-9375-x

[B2] Ackerman R. Goldsmith M. (2011). Metacognitive regulation of text learning: on screen versus on paper. J. Exp. Psychol. Appl. 17, 18–32. 10.1037/a0022086 21443378

[B3] Andrews-Hanna J. R. Smallwood J. Spreng R. N. (2014). The default network and self–generated thought: component processes, dynamic control, and clinical relevance. Ann. N. Y. Acad. Sci. 1316, 29–52. 10.1111/nyas.12360 24502540 PMC4039623

[B4] Barr N. Pennycook G. Stolz J. A. Fugelsang J. A. (2015). The brain in your pocket: evidence that smartphones are used to supplant thinking. Comput. Hum. Behav. 48, 473–480. 10.1016/j.chb.2015.02.029

[B5] Berman M. G. Jonides J. Kaplan S. (2008). The cognitive benefits of interacting with nature. Psychol. Sci. 19, 1207–1212. 10.1111/j.1467-9280.2008.02225.x 19121124

[B6] Berto R. Massaccesi S. Pasini M. (2008). Do eye movements measured across high and low fascination photographs differ? Addressing Kaplan's fascination hypothesis. J. Environ. Psychol. 28, 185–191. 10.1016/j.jenvp.2007.11.004

[B7] Billieux J. Maurage P. Lopez-Fernandez O. Kuss D. J. Griffiths M. D. (2015). Can disordered mobile phone use be considered a behavioral addiction? An update on current evidence and a comprehensive model for future research. Curr. Addict. Rep. 2, 156–162. 10.1007/s40429-015-0054-y

[B8] Bratman G. N. Hamilton J. P. Daily G. C. (2012). The impacts of nature experience on human cognitive function and mental health. Ann. N. Y. Acad. Sci. 1249, 118–136. 10.1111/j.1749-6632.2011.06400.x 22320203

[B9] Brown T. H. Mbati L. S. (2015). Mobile learning: moving past the myths and embracing the opportunities. Int. Rev. Res. Open Distributed Learn. 16, 115–135. 10.19173/irrodl.v16i2.2071

[B10] Christoff K. Irving Z. C. Fox K. C. Spreng R. N. Andrews-Hanna J. R. (2016). Mind-wandering as spontaneous thought: a dynamic framework. Nat. Rev. Neurosci. 17, 718–731. 10.1038/nrn.2016.113 27654862

[B11] Chun M. M. Golomb J. D. Turk-Browne N. B. (2011). A taxonomy of external and internal attention. Annu. Rev. Psychol. 62, 73–101. 10.1146/annurev.psych.093008.100427 19575619

[B12] Colzato L. S. Hommel B. (2017). Meditation, in Theory-Driven Approaches to Cognitive Enhancement, eds Colzato L. (Cham: Springer), 226–235.

[B13] Colzato L. S. Ozturk A. Hommel B. (2012). Meditate to create: the impact of focused-attention and open-monitoring training on convergent and divergent thinking. Front. Psychol. 3:116. 10.3389/fpsyg.2012.00116 22529832 PMC3328799

[B14] Colzato L. S. Szapora A. Lippelt D. Hommel B. (2017). Prior meditation practice modulates performance and strategy use in convergent-and divergent-thinking problems. Mindfulness 8, 10–16. 10.1007/s12671-014-0352-9

[B15] Dadvand P. Nieuwenhuijsen M. J. Esnaola M. Forns J. Basagaña X. Alvarez-Pedrerol M.. (2015). Green spaces and cognitive development in primary schoolchildren. Proc. Natl. Acad. Sci. U.S.A. 112, 7937–7942. 10.1073/pnas.1503402112 26080420 PMC4491800

[B16] Davis N. Gatersleben B. (2013). Transcendent experiences in wild and manicured settings: the influence of the trait “connectedness to nature.” Ecopsychology 5, 92–102. 10.1089/eco.2013.0016

[B17] Diamond A. (2011). Biological and social influences on cognitive control processes dependent on prefrontal cortex. Prog. Brain Res. 189, 319–339. 10.1016/B978-0-444-53884-0.00032-4 21489397 PMC4103914

[B18] Diamond A. (2013). Executive functions. Ann. Rev. Psychol. 64, 135–168. 10.1146/annurev-psych-113011-143750 23020641 PMC4084861

[B19] Dong G. Potenza M. N. (2017). Internet searching and memory processing during a recollection fMRI task: evidence from pseudo recollected trials. J. Technol. Behav. Sci. 1, 32–36. 10.1007/s41347-016-0002-2

[B20] Duarte Torres S. Weber I. Hiemstra D. (2014). Analysis of search and browsing behavior of young users on the web. ACM Trans. Web. 8:7 10.1145/2555595

[B21] Elhai J. D. Vasquez J. K. Lustgarten S. D. Levine J. C. Hall B. J. (2017). Proneness to boredom mediates relationships between problematic smartphone use with depression and anxiety severity. Soc. Sci. Comput. Rev. 1–14. 10.1177/0894439317741087

[B22] Engle R. W. (2002). Working memory capacity as executive attention. Curr. Dir. Psychol. Sci. 11, 19–23.

[B23] Heersmink R. (2016). The internet, cognitive enhancement, and the values of cognition. Minds Machines 26, 389–407. 10.1007/s11023-016-9404-3

[B24] Holloway D. Green L. Livingstone S. (2013). Zero to Eight. Young Children and Their Internet Use. London: LSE; EU Kids Online.

[B25] Hommel B. Colzato L. S. (2017). Meditation and metacontrol. J. Cogn. Enhancement 1, 115–121. 10.1007/s41465-017-0017-4 PMC7089713 32226918

[B26] Howell A. J. Dopko R. L. Passmore H. A. Buro K. (2011). Nature connectedness: associations with well-being and mindfulness. Pers. Individ. Dif. 51, 166–171. 10.1016/j.paid.2011.03.037

[B27] James W. (1892). Text-Book of Psychology. London: Macmillan.

[B28] Kahn P. S. Jr. Severson R. L. Ruckert J. H. (2009). The human relation with nature and technological nature. Curr. Dir. Psychol. Sci. 18, 37–42. 10.1111/j.1467-8721.2009.01602.x

[B29] Kaplan S. (1995). The restorative benefits of nature: toward an integrative framework. J. Environ. Psychol. 15, 169–182. 10.1016/0272-4944(95)90001-2

[B30] Kaplan S. Berman M. G. (2010). Directed attention as a common ressource for executive functioning and self-regulation. Perspect. Psychol. Sci. 5, 43–57. 10.1177/1745691609356784 26162062

[B31] Keinänen M. (2016). Taking your mind for a walk: a qualitative investigation of walking and thinking among nine Norwegian academics. High. Educ. 71, 593–605. 10.1007/s10734-015-9926-2

[B32] Kuo M. Browning M. H. Penner M. L. (2017). Do lessons in nature boost subsequent classroom engagement? Refueling students in flight. Front. Psychol. 8:2253. 10.3389/fpsyg.2017.02253 29354083 PMC5758746

[B33] Lauterman T. Ackerman R. (2014). Overcoming screen inferiority in learning and calibration. Comput. Hum. Behav. 35, 455–463. 10.1016/j.chb.2014.02.046

[B34] LeBlanc A. G. Chaput J. P. (2017). Pokémon GO: a game changer for the physical inactivity crisis? Prev. Med. 101, 235–237. 10.1016/j.ypmed.2016.11.012 27856340

[B35] Lebuda I. Zabelina D. L. Karwowski M. (2016). Mind full of ideas: a meta-analysis of the mindfulness–creativity link. Pers. Individ. Dif. 93, 22–26. 10.1016/j.paid.2015.09.040

[B36] Lee Y. K. Chang C. T. Lin Y. Cheng Z. H. (2014). The dark side of smartphone usage: psychological traits, compulsive behavior and technostress. Comput. Hum. Behav. 31, 373–383. 10.1016/j.chb.2013.10.047

[B37] Leong L. Y. C. Fischer R. McClure J. (2014). Are nature lovers more innovative? The relationship between connectedness with nature and cognitive styles. J. Environ. Psychol. 40, 57–63. 10.1016/j.jenvp.2014.03.007

[B38] Li J. Lepp A. Barkley J. E. (2015). Locus of control and cell phone use: implications for sleep quality, academic performance, and subjective well-being. Comput. Hum. Behav. 52, 450–457. 10.1016/j.chb.2015.06.021

[B39] Lutz A. Slagter H. A. Dunne J. D. Davidson R. J. (2008). Attention regulation and monitoring in meditation. Trends Cogn. Sci. 12, 163–169. 10.1016/j.tics.2008.01.005 18329323 PMC2693206

[B40] Major L. Haßler B. Hennessy S. (2017). Tablet use in schools: impact, affordances and considerations, in Handbook on Digital Learning for K-12 Schools, eds Marcus-Quinn A. Hourigan T. (Cham: Springer International Publishing), 115–128.

[B41] Matsuoka R. H. (2010). Student performance and high school landscapes: examining the links. Landsc. Urban Plan. 97, 273–282. 10.1016/j.landurbplan.2010.06.011

[B42] Mbati L. (2017). Creating awareness around rhizomatic principles in mlearning: a means to improving practice. Int. J. Mobile Blended Learn. 9, 74–87. 10.4018/IJMBL.2017040105

[B43] Misra S. Cheng L. Genevie J. Yuan M. (2016). The iPhone effect the quality of in-person social interactions in the presence of mobile devices. Environ. Behav. 48, 275–298. 10.1177/0013916514539755

[B44] Norris C. A. Soloway E. (2015). Mobile technology in 2020: predictions and implications for K-12 education. Educ. Technol. 55, 12–19.

[B45] Ohly H. Gentry S. Wigglesworth R. Bethel A. Lovell R. Garside R. (2016). A systematic review of the health and well-being impacts of school gardening: synthesis of quantitative and qualitative evidence. BMC Public Health 16, 286. 10.1186/s12889-016-2941-0 27015672 PMC4807565

[B46] Oppezzo M. Schwartz D. L. (2014). Give your ideas some legs: the positive effect of walking on creative thinking. J. Exp. Psychol. Learn. Mem. Cogn. 40, 1142–1152. 10.1037/a0036577 24749966

[B47] Piccoli T. Valente G. Linden D. E. Re M. Esposito F. Sack A. T.. (2015). The default mode network and the working memory network are not anti-correlated during all phases of a working memory task. PLoS ONE 10:e0123354. 10.1371/journal.pone.0123354 25848951 PMC4388669

[B48] Posner M. I. Rothbart M. K. Tang Y. (2013). Developing self-regulation in early childhood. Trends Neurosci. Educ. 2, 107–110. 10.1016/j.tine.2013.09.001 24563845 PMC3927309

[B49] Przybyliski A. K. Weinstein N. (2013). Can you connect with me now? How the presence of mobile communication technology influences face-to-face conversation quality. J. Soc. Pers. Relat. 30, 237–246. 10.1177/0265407512453827

[B50] Roberts J. A. Pullig C. Manolis C. (2015). I need my smartphone: a hierarchical model of personality and cell-phone addiction. Pers. Individ. Dif. 79, 13–19. 10.1016/j.paid.2015.01.049

[B51] Rosen L. D. Whaling K. Rab S. Carrier L. M. Cheever N. A. (2013). Is Facebook creating “iDisorders”? The link between clinical symptoms of psychiatric disorders and technology use, attitudes and anxiety. Comput. Hum. Behav. 29, 1243–1254. 10.1016/j.chb.2012.11.012

[B52] Ruiz-Ariza A. Casuso R. A. Suarez-Manzano S. Martínez-López E. J. (2018). Effect of augmented reality game Pokémon GO on cognitive performance and emotional intelligence in adolescent young. Comput. Educ. 116, 49–63. 10.1016/j.compedu.2017.09.002

[B53] S Colzato L. Szapora A. Pannekoek J. N. Hommel B. (2013). The impact of physical exercise on convergent and divergent thinking. Front. Hum. Neurosci. 7:824. 10.3389/fnhum.2013.00824 24348370 PMC3845014

[B54] Schilhab T. (2017a). Impact of iPads on break-time in primary schools—a Danish context. Oxf. Rev. Educ. 43, 261–275. 10.1080/03054985.2017.1304920

[B55] Schilhab T. (2017b). Adaptive smart technology use: the need for meta-self-regulation. Front. Psychol. 8:298. 10.3389/fpsyg.2017.00298. 28303112 PMC5332409

[B56] Schilhab T. (2017c). Derived Embodiment in Abstract Language. Cham: Springer 10.1007/978-3-319-56056-4

[B57] Schilhab T. (2018). Can your child's phone bring them closer to nature? ScienceNordic. Available online at: http://sciencenordic.com/can-your-child%E2%80%99s-phone-bring-them-closer-nature (Accessed January 10, 2018).

[B58] Smallwood J. Schooler J. W. (2006). The restless mind. Psychol. Bull. 132, 946–958. 10.1037/0033-2909.132.6.946 17073528

[B59] Sood A. Jones D. T. (2013). On mind wandering, attention, brain networks and meditation. Explore 9, 136–141. 10.1016/j.explore.2013.02.005 23643368

[B60] Sparrow B. Liu J. Wegner D. M. (2011). Google effects on memory: cognitive consequences of having information at our fingertips. Science 333, 776–778. 10.1126/science.1207745 21764755

[B61] Tang Y. Y. Posner M. I. (2009). Attention training and attention state training. Trends Cogn. Sci. 13, 222–227. 10.1016/j.tics.2009.01.009 19375975

[B62] Tanis M. Beukeboom C. J. Hartmann T. Vermeulen I. E. (2015). Phantom phone signals: an investigation into the prevalence and predictors of imagined cell phone signals. Comput. Hum. Behav. 51, 356–362. 10.1016/j.chb.2015.04.039

[B63] Traxler J. Wishart J. (2011). Making Mobile Learning Work: Case Studies of Practice. Bristol: ESCalate, HEA Subject Centre for Education, University of Bristol.

[B64] Turkle S. (2015). Reclaiming Conversation: The Power of Talk in a Digital Age. Penguin. New York, NY: Penguin Books.

[B65] Ward A. F. Duke K. Gneezy A. Bos M. W. (2017). Brain drain: the mere presence of one's own smartphone reduces available cognitive capacity. J. Assoc. Consum. Res. 2, 140–154. 10.1086/691462

[B66] Wilson M. (2002). Six views on embodied cognition. Psychon. Bull. Rev. 9, 625–635. 10.3758/BF03196322 12613670

[B67] Zhou Y. Zhang Y. Hommel B. Zhang H. (2017). The impact of bodily states on divergent thinking: evidence for a control-depletion account. Front. Psychol. 8:1546. 10.3389/fpsyg.2017.01546 29033862 PMC5626876

